# Carbon information disclosure quality, greenwashing behavior, and enterprise value

**DOI:** 10.3389/fpsyg.2022.892415

**Published:** 2022-08-04

**Authors:** Qilin Cao, Yunhuan Zhou, Hongyu Du, Mengxi Ren, Weili Zhen

**Affiliations:** ^1^Business School, Sichuan University, Chengdu, China; ^2^Department of Biostatistics and Bioinformatics, Duke University, Durham, NC, United States; ^3^Management School, Sichuan Agricultural University, Chengdu, China

**Keywords:** carbon information disclosure, greenwashing behavior, enterprise value, dual carbon goal, corporate social responsibility

## Abstract

As global warming becomes increasingly prominent, countries worldwide advocate for a low-carbon economy to cope with the pressure to reduce greenhouse gas emissions. The Chinese government has proposed a “dual carbon” goal of peaking carbon emissions by 2030 and becoming carbon neutral by 2060. The disclosure of carbon information by Chinese enterprises has attracted widespread attention from society. This study selects the constituents of the Social Responsibility Index of China Shanghai Stock Exchange from 2016 to 2020 as samples to empirically analyze the relationship between the level of carbon information disclosure and corporate value, and the moderating effect of greenwashing behavior. Results indicated that the quality of carbon disclosure is positively correlated with the enterprise value. Greenwashing behavior promotes the positive impact of carbon disclosure quality on enterprise value in the short run, but this promoting effect fades in the long run. We further found that the carbon information disclosure of non-heavy-pollution enterprises has a more obvious positive impact on enterprise value than that of heavily polluting enterprises. Additionally, the positive impact of carbon information disclosure on enterprise value is more visible among enterprises in a good legal environment than those in a poor legal environment. This study enriches the relevant literature on carbon information disclosure and enterprise “greenwashing” behavior and has practical significance for promoting China’s low-carbon development in the context of ecological civilization and improving the enthusiasm for the quality of enterprise carbon information disclosure.

## Introduction

Natural disasters and public health events caused by global warming have occurred frequently worldwide (Marino et al., 2016). Extreme weather, such as typhoons and tsunamis, has occurred continuously worldwide, posing a serious threat to the survival and development of humanity (Todea et al., 2013). In deteriorating living environments, there is a global consensus to curb greenhouse gas emissions and achieve the sustainable development of human beings. Most countries believe that a low-carbon economy is an effective solution to the problem of climate change and has become the development trend of the current global economy (Ionescu, 2021; He et al., 2022).

As one of the largest carbon emitters in the world, China is also a participant in constructing a global ecological civilization and actively demonstrates its responsibility as a major country in the global carbon reduction course (He et al., 2022). In 2015, at the Paris Climate Conference, China committed to reduce carbon emissions per unit of GDP by 60%–65% from 2005 to 2030 (Tollefson, 2016). China launched a national carbon trading market covering major industries in 2017. In 2021, the State Council of China formulated the 14th Five-Year Plan for Energy Conservation and Emission Reduction to promote the all-around green transformation of economic and social development and help achieve the “dual carbon” goal. China’s economic development goals have shifted from high-speed growth to high-quality development. A series of policies successively issued by the Chinese government reflect the high importance of carbon emission reduction.

Being cells of the national economy, enterprises are the main sources of carbon emissions (He et al., 2022). As an important way for enterprises to show their carbon emission data and low-carbon behavior to stakeholders, carbon information disclosure has attracted increasing attention from academia and the public. Enterprises disclose social responsibility reports or sustainable development reports to the public to show that they actively undertake social responsibilities, such as environmental protection. Conversely, enterprises can use disclosed carbon information to analyze their environmental risks, avoid risks, seize opportunities, and improve enterprise value (Yan and Chen, 2017). Disclosed carbon information can be an important indicator for stakeholders in evaluating the effectiveness of corporate carbon emission reduction and serve as a window to convey signals of corporate low-carbon behavior. In this context, it is of great significance to study the impact of carbon information disclosure on enterprise value.

However, China’s current carbon enterprises still belong to the category of voluntary disclosure of information. The content and methods of carbon disclosure are not standardized, and information disclosure becomes formal. Under the natural opportunism tendency of enterprises and asymmetric information in the green market, enterprises may encounter adverse selection and moral hazards to meet external demand and obtain higher returns. They may adopt greenwashing strategies regarding disclosure content and depth to whitewash their environmental performance (Huang et al., 2019). The greenwashing phenomenon is a new type of unethical business behavior in response to environmental regulations and green management practices (Laufer, 2003). It is a response mode of social responsibility that adapts to form without making fundamental changes. Greenwashing is a means for enterprises to meet the needs of legitimacy and interact positively with stakeholders to establish a good corporate image (Huang, 2020). Greenwashing behavior also has negative effects on enterprises. After exposure to this behavior, the cumulative excess return rate of an enterprise is significantly negative (Du, 2015), which also influences its green brand effect and corporate reputation, causing significant losses (Akturan, 2018). Existing studies have investigated the impact of greenwashing behavior on green buying (Akturan, 2018; Zhang et al., 2018) and corporate financial performance (Testa et al., 2018). However, few studies have discussed the relationship between greenwashing and enterprise value, and the moderating effect of greenwashing on the relationship between carbon information disclosure and enterprise value.

We used the Shanghai Stock Exchange Social Responsibility Index in selecting our sample enterprises. This index is a sample stock comprising the top 100 enterprises with social contribution value per share of corporate governance on the Shanghai Stock Exchange. It is composed of companies that have performed well in fulfilling their social responsibilities as sample stocks, and plays a leading role in disclosing the social responsibility information of listed companies. Research on carbon information disclosure and greenwashing behavior can encourage and promote other listed companies to actively fulfill their social responsibilities, deepen their environmental awareness, and enrich the sample selection for future research (Wang and Jin, 2013).

This study analyzed the impact of carbon disclosure and greenwashing behavior on enterprise value. It discusses the moderating effect of greenwashing behavior on the relationship between carbon disclosure and enterprise value. It further discusses the impact of industry and legal environment heterogeneities on the relationship between carbon disclosures and enterprise value. Existing literature has discussed the impact of carbon information disclosure on an enterprise value. However, few studies have focused on the greenwashing behavior of enterprises and applied it to empirical research. This study extends the research on the factors influencing enterprise value and enriches the theoretical research on carbon information disclosure and greenwashing. It innovates by using the specific characteristics and factors of the data and principal component analysis. It also builds the carbon disclosure quality of the evaluation system and introduces a floating green behavior recognition model applied to empirical research, which provides a new idea for future studies of carbon disclosure quality and floating green behavior indicators. In addition, the moderating effect of greenwashing is discussed from both short-term and long-term perspectives. A heterogeneity test of the industry and legal environment was also conducted in this study. The difference in the impact of carbon information disclosure on enterprise value for heavily polluting versus non-heavily polluting enterprises and for enterprises in good legal environments versus those in poor legal environments is investigated. Furthermore, this study has practical significance; it provides empirical evidence for enterprises to actively respond to the call for “double carbon” and operate low-carbon businesses to improve their corporate value. It provides a reference for government regulatory authorities and other stakeholders in evaluating the quality of carbon information disclosure and supervising enterprises’ greenwashing behaviors. It also regulates management’s carbon information disclosure behavior from an organizational psychology perspective to enhance corporate value.

The remainder of this paper is organized as follows: Section “Literature Review and Hypothesis” presents the literature review and hypotheses; Section “Materials and Methods” presents the materials and methods and the empirical research design; Section “Results” presents the results and analyzes the empirical results; and Section “Conclusion, Revelations, and Limitations” provides the conclusion, revelations, and limitations of the study.

## Literature review and hypothesis

### Carbon information disclosure quality and enterprise value

The level of carbon information disclosure affects enterprise value, and domestic and foreign scholars have drawn different conclusions from different research perspectives. Enterprises with more carbon inputs or significant carbon performance tend to disclose more carbon information. Therefore, compared to enterprises with a lower level of carbon information disclosure, enterprises with a higher level of carbon information disclosure tend to take the initiative in carbon management and invest more capital, technology, and other resources. This affects the resource input of enterprises in business activities and other aspects, thereby reducing short-term performance to a certain extent (Liu et al., 2021). Additionally, suppose that illegal pollution is included in carbon information disclosed by enterprises. In this case, intangible pressure will also be brought to enterprises, making them passively invest more costs and resources for carbon control. This will also be detrimental to short-term performance and not conducive to enhancing the short-term value of the enterprise.

However, academics have arrived at the opposite conclusion in the long term. According to social responsibility theory, enterprises must undertake specific social responsibilities while creating value for themselves. The excellence of corporate governance lies in its ability to take on corporate social responsibility and thus promote growth (Akram et al., 2020). Environmental protection and reduction of carbon emissions are critical components of social responsibility. Global warming is becoming an increasingly serious issue. People increasingly focus on enterprise information disclosure of carbon. Carbon information disclosure includes strategy, governance, and measures to help stakeholders effectively make decisions. Meanwhile, when investors consciously and actively take responsibility for a good corporate image, it increases an enterprise’s reputation (Horiuchi et al., 2009). As an essential intangible asset, corporate reputation can bring capital premiums to an enterprise. This can enhance the confidence of many investors, attract more investments, and improve enterprise value in the long run (Le et al., 2020).

According to organizational legitimacy theory, if an organization wants to survive for a long time, its behavior must conform to the values and norms recognized by the public. The carbon information disclosure of enterprises must conform to the corresponding legal standards and codes of conduct. This is conducive to enhancing the organization’s legitimacy, alleviating the adverse impact of negative news on enterprises, gaining public recognition and support, and improving enterprises’ long-term value (Liu et al., 2021).

Additionally, from the perspective of signal transmission theory, enterprises’ disclosure of internal information alleviates the information asymmetry between enterprises and investors. It enhances the recognition and trust of investors in enterprises, thus increasing investment and improving enterprise value (Li et al., 2016). Enterprises that disclose higher-quality carbon information can convey a more competitive advantage signal to investors and other information users, establish a good corporate image, and thus stimulate investors’ willingness to buy their stocks. This increases stock liquidity and promotes value creation. Thus, hypothesis one is proposed as follows:

*H1*: The quality of carbon information disclosure is positively correlated with enterprise value.

### Carbon information disclosure quality, greenwashing behavior, and enterprise value

#### Short-term effects of greenwashing

Greenwashing enterprises can conduct impression management when they voluntarily disclose environmental information. Scholars at home and abroad have found through their research on corporate annual reports that enterprises influence information recipients’ understanding of the company through self-serving attribution, manipulation of readability and comprehensibility, charts, and color rendering (Brennan et al., 2009). Greenwashing enterprises often use word games to make superficial appearances and use vague, ambiguous, and symbolic language to “whitewash” their environmental performance (Walker and Wan, 2012). From an organizational psychology perspective, senior executives’ psychology also affects enterprises’ carbon information disclosure. Senior executives, as corporate managers, are motivated to selectively disclose information that is beneficial to their interests (Lu et al., 2017). Enterprises often implement incentive measures for their management. This increases the possibility that senior executives choose to disclose information beneficial to their operations and management for their interests while deliberately concealing the information affecting the enterprise. From the viewpoint of new institutional economics, information asymmetry and human limited rationality provide favorable opportunities for enterprises’ greenwashing behavior, which urges them to try to establish a responsible social image through lying and deception (Xiao et al., 2013). Stakeholders are the inferior side of information, and their cognition of products mainly comes from advertising and their own experience (Leonidou et al., 2013), which also gives enterprises the opportunity to adopt greenwashing behavior to seek benefits for themselves. Carbon information is important non-financial information for a company, while disclosure information under greenwashing behavior shows more positive performance of carbon emission reduction responsibility and better environmental performance (Delmas and Burbano, 2011). Greenwashing behavior can improve a company’s social recognition and stakeholders’ expectations of the company’s future behavior and performance, promote investor optimism (Gatti et al., 2021), and reduce expected risks, thus reducing the cost of equity capital for the enterprise. This improves the expected cash flow of the enterprise and promotes an improvement in enterprise value. In the short run, the greenwashing behavior of enterprises has not been perceived by the public. Through low-cost greenwashing, enterprises can avoid punishment and even improve their profits (Wang et al., 2015). Therefore, in the short run, this sends out a seemingly more transparent corporate signal, reflecting the enterprise’s investment in maximizing stakeholders’ interests. Greenwashing behavior can improve an enterprise’s social recognition, promote investor optimism, and reduce expected risks, thus reducing the cost of equity capital of the enterprise. This improves the expected cash flow of the enterprise and promotes an improvement in enterprise value. Based on this, hypothesis two is proposed as follows:

*H2*: In the short run, greenwashing behavior promotes the positive effect of carbon information disclosure quality on enterprise value.

#### Long-term effects of greenwashing

Many researchers have studied the negative effects of greenwashing. Walker and Wan’s (2012) study of two heterogeneous environmental responsibility response strategies, true green and greenwashing, found that although the true green strategy does not correlate with corporate financial performance, the “hypocritical” greenwashing strategy negatively impacts financial performance. Matejek and Goessling (2014) conducted a case study on BP suspected of greenwashing after an oil spill in the Gulf of Mexico. They found that BP’s stock price dropped by approximately one-third after exposure to greenwashing and its market value evaporated by approximately 70 billion dollars. Wu et al. (2020) regard the Corporate Social Report (CSR) as a marketing gimmick. They developed a game-theoretic model of CSR investment to examine how information transparency affects a firm’s strategies and social welfare and further identified the positive and negative aspects of greenwashing. Yu et al. (2020) pointed out that because the information on environmental social governance (ESG) disclosed by enterprises is not audited, the greenwashing behavior of enterprises may become an obstacle to incorporating ESG factors into investment decisions.

Greenwashing is a pseudo-social responsibility behavior. Some scholars have suggested that pseudo-social responsibility behaviors may cause a loss of overall social welfare or fail to promote social welfare (Fassin and Buelens, 2011). Even in the current institutional environment, the risk of corporate pseudo-social responsibility behavior being discovered is low; however, in the long run, it will eventually be exposed, and the false responsible image created by enterprises through pseudo-social responsibility behavior will eventually collapse (Xiao et al., 2013). Once the greenwashing behavior of enterprises is exposed, it will be regarded as “socially irresponsible” behavior, which will have negative effects on enterprises from multiple aspects, including punishment of enterprises by the capital market, resulting in a decline in shareholder wealth (Frooman, 1997). Janney and Gove (2011) believe that when the fake social responsibility of enterprises is perceived by consumers, they will not only feel betrayed, but also want to stay away from such enterprises. In the long run, brand loyalty will be lost, and customers will inform others of the pseudo-social responsibility behavior of enterprises, which will damage the reputation of enterprises. Furthermore, it can lead to a loss of confidence among consumers, investors, and non-governmental organizations (Painter-Morland, 2006; Jahdi and Acikdilli, 2009; Lyon and Montgomery, 2015). Greenwashing may create a short-term gain for deceptive companies; however, in the long run, the entire green market will experience a fall (Polonsky et al., 2010). If the environmental, social, and governance information disclosed by enterprises is unreliable, the greenwashing behavior of enterprises may become an obstacle to incorporating environmental, social, and governance factors into investment decisions (Yu et al., 2020). Over time, greenwashing damages investor confidence and provokes negative market feedback (Yang et al., 2020). At the same time, it inhibits investors’ optimism, and the positive promotion effect of carbon information disclosure on enterprise value fades with it. Based on this, hypothesis three is proposed as follows:

*H3*: In the long run, the positive effect of greenwashing behavior on promoting the quality of carbon information disclosure on enterprise value fades.

## Materials and methods

### Sample and data sources

Unlike the semi-mandatory disclosure policy of social responsibility information disclosure, carbon information disclosure is voluntary. Hence, this study attempted to select samples with fewer missing values. According to Wang and Jin (2013), Shanghai Stock Exchange Social Responsibility Index is a sample stock comprising the top 100 enterprises with social contribution value per share of corporate governance on the Shanghai Stock Exchange. It is generally believed that information disclosure by constituent companies is relatively comprehensive. By contrast, carbon information disclosure, which is part of environmental disclosure in social responsibility disclosure, has a lower missing value. However, considering that 2016–2020 was the implementation stage of China’s 13th Five-Year Plan, a study on implementing energy conservation and emission reduction during this period will provide valuable experience and lessons for the performance of green development goals during the 14th Five-Year Plan.

Therefore, data from 100 enterprises in the Social Responsibility Index of the Shanghai Stock Exchange from 2016 to 2020 were selected as our research samples. Since the financial industry’s participation in the carbon trading mechanism is different from the carbon emission reduction method and other industries, such as the traditional manufacturing industry and agriculture, its financial information cannot meet research needs. A total of 350 data samples were collected, after excluding financial enterprises and enterprises with missing data. Carbon and greenwashing information are mainly from corporate social responsibility and sustainability reports, while other data are from the China Stock Market and Accounting Research (CSMAR) database.

### Dependent variable

The dependent variable was enterprise value (V). Li et al. (2017a, 2017b) consider Tobin’s Q value considering the circumstances of the present and future of the enterprise as the company’s market value and the ratio of the current replacement cost. It comprehensively embodies the present and future value of an enterprise’s potential profitability and is more apt to measure enterprise value. Therefore, this study chooses Tobin’s Q value to calculate the enterprise value.

### Independent variables

#### Carbon information disclosure quality

The independent variable is the quality of carbon information disclosure (CID). This study followed the practices of Li et al. (2017a,b, 2019). Based on the four dimensions of carbon information disclosure in corporate reports (as shown in Table 1), a preliminary score is given to corporate social responsibility or sustainable development reports. Based on the scoring results, a quality evaluation model for carbon information disclosure was innovatively constructed. By using this model, we can obtain a more scientific quality index. This is convenient for studying the relationship between the carbon information disclosure quality of the sample enterprises under the influence of greenwashing and enterprise value.

**Table 1:** Carbon information disclosure evaluation index system.

First-level variables	Second-level variables	Index scoring standard	Explanation
Timeliness	Disclosure time of the report	After annual report disclosure = 0; annual report disclosure or subsequent = 1	We need to check whether the carbon information disclosure (social responsibility report) is released in a reasonable time. Assign a value of 0 if the disclosure time is after the annual report disclosure; assign a value of 1, if it is before the annual report disclosure.
Reliability	Report collection process system	No system description = 0; relevant greenhouse gas description or calculation method = 1; detailed and complete process system description = 2	We need to check the description in the carbon information collection process system. Assign a value of 0 if there is no system introduction; assign a value of 1 if there is related greenhouse gas device description or calculation method introduction; assign a value of 2 if there is detailed and complete process system introduction.
	Examination and verification of evidence	No inspection = 0; social responsibility report authentication = 1; specialized carbon information disclosure authentication = 2	We need to check whether the disclosed carbon information is independently verified by a third party. If there is no verification or authentication of the carbon information disclosure, assign a value of 0; if there is verification of social responsibility report (carbon information is involved in the report, otherwise, it will be deemed as no verification), assign a value of 1; if there is special verification of the carbon information disclosure, assign a value of 2.
Comprehensibility	Picture and text description	None of the three = 0; only one of the three = 1; text + data = 2; synthesis of the three = 3	We need to check the balance of the use of text, data, and chart in the carbon disclosure information. If none, assign a value of 0; if there are only one of three, assign a value of 1; if there is text and data, assign a value of 2; if all three are combined, assign a value of 3.
	Professional term	No technical terms = 0; there are technical terms and no explanation = 1; there are technical terms with explanatory notes = 2	We need to check whether there are technical terms and their explanations in the carbon information disclosed (such as carbon dioxide equivalent, carbon sequestration). If there is no technical term, assign a value of 0; if there is a technical term but no explanation, assign a value of 1; if there is a technical term with an explanation, assign a value of 2.
Comparability	Quantitative standard of carbon accounting	No calculation methods and data = 0; there are popular standardized specific data = 1; there are calculation methods and specific data = 2	We need to check whether the carbon information accounting quantitative standards are unified. If no calculation method and data are available, assign a value of 0; if there is specific data of standardization, assign a value of 1; if there is calculation method and specific data, assign a value of 2.
Completeness	Emission reduction strategy	No explanation = 0; simple description = 1; detailed description = 2	We need to judge whether there is a description of a carbon emission reduction in the strategic plan. If no indication is given, assign a value of 0; if stated simply, assign a value of 1; if specified, assign a value of 2.
	Emission reduction target	Non-emission reduction target note = 0; qualitative description plan only = 1; qualitative + quantitative description plan = 2	We need to judge whether a carbon emission reduction target is disclosed in the report. If there is no description of the emission reduction target, assign a value of 0; if there is only a qualitative description of the plan, assign a value of 1; if there is a qualitative and quantitative description of the plan, assign a value of 2.
	Emission reduction management	Unreduced emission management note = 0; brief description = 1; detailed description = 2	We need to look at the establishment of functional emission reduction organizations, emission reduction management systems and other measures related to carbon emission reduction. If there is no emission reduction management description, assign a value of 0; if there is a simple description, assign a value of 1; if there is a detailed description, assign a value of 2.
	Emission reduction risk	No emission reduction risk note = 0; brief description = 1; detailed description = 2	We need to check whether the report contains descriptions of emission reduction risks such as non-emission reduction risks caused by government regulations, business risks caused by climate change, or possible loss of economic benefits caused by emission reduction. If there is no risk description for emission reduction, assign a value of 0; if there is a simple description, assign a value of 1; if there is a detailed description, assign a value of 2.
	Emission reduction input	No emission reduction input description = 0; qualitative description only = 1; qualitative + quantitative description = 2	We need to check whether the report shows the technical improvement and project investment for carbon emission reductions and the emission discharge fees and fines paid. If there is no description of emission reduction input, assign a value of 0, if there is only a qualitative description, assign a value of 1; if there is a qualitative and quantitative description, assign a value of 2.
	Emission reduction subsidy	No explanation = 0; simple description = 1; detailed description = 2	We need to check whether the enterprise received any emission reduction subsidies or incentives from the government and other carbon information disclosure. If no indication is given, assign a value of 0; if stated simply, assign a value of 1; if specified, assign a value of 2.
	Emission reduction accounting	No accounting note = 0; qualitative description only = 1; qualitative + quantitative description = 2	We need to check whether the enterprise has disclosed carbon information such as accounting method, tons of energy saving and tons of emission reduction. If there is no description of the emission reduction target, assign a value of 0; if there is only a qualitative description of the plan, assign a value of 1; if there is a qualitative and quantitative description of the plan, assign a value of 2.
	Emission reduction performance	No explanation = 0; only qualitative description = 1; qualitative + quantitative description = 2	We need to check whether there is any economic benefit, environmental benefit, social benefit, honor, or other carbon information disclosure generated by emission reduction. If there is no description of the emission reduction target, assign a value of 0; if there is only a qualitative description of the plan, assign a value of 1; if there is a qualitative and quantitative description of the plan, assign a value of 2.

##### Data introduction

As shown in Table 1, we have four first-level variables and 14 s-level variables. They are “report disclosure time,” “report collection process,” “review and verification,” “graphic description,” “professional terms,” “carbon accounting quantification standard,” “emission reduction strategy,” “emission reduction target,” “emission reduction management,” “emission reduction risk,” “emission reduction input,” “emission reduction subsidy,” “emission reduction accounting,” and “emission reduction performance.” All the variables above are categorical variables, and 13 of them have only three categories: 0, 1, and 2. The graphic description had four categories: 0, 1, 2, and 3. Because there is no given variable of carbon information disclosure in this data (it is not easy to obtain accurate values because it has excellent subjectivity and instability), we use unsupervised learning to analyze the data structure and then establish a formula for the carbon information disclosure model.

##### Correlation analysis

First, we tested the correlation analysis of the 14 s-level variables. Two groups of variables with high correlation should have high collinearity. The approach used to analyze the correlation coefficient matrix is as follows: When the correlation coefficient of the two variables was higher than 0.7, a high correlation was observed. Meanwhile, the variables should be deleted or the sample size should be increased. The two groups of data with a correlation coefficient lower than 0.7 are not considered to be strongly correlated. K1–K14 represent 14 secondary variables, and their correlation coefficient matrices are listed in Table 2.

**Table 2:** Correlation matrix of second-level variables.

	$k_{1}$	$k_{2}$	$k_{3}$	$k_{4}$	$k_{5}$	$k_{6}$	$k_{7}$	$k_{8}$	$k_{9}$	$k_{\mathrm{10}}$	$k_{\mathrm{11}}$	$k_{\mathrm{12}}$	$k_{\mathrm{13}}$	$k_{\mathrm{14}}$
$k_{1}$	1													
$k_{2}$	−0.15	1												
$k_{3}$	−0.08	0.37	1											
$k_{4}$	−0.11	0.44	0.49	1										
$k_{5}$	−0.10	0.43	0.16	0.27	1									
$k_{6}$	−0.06	0.59	0.36	0.59	0.56	1								
$k_{7}$	−0.14	0.3	0.31	0.36	0.32	0.44	1							
$k_{8}$	−0.07	0.33	0.19	0.19	0.34	0.41	0.56	1						
$k_{9}$	−0.08	0.46	0.31	0.46	0.29	0.45	0.41	0.51	1					
$k_{\mathrm{10}}$	−0.04	0.17	0.12	0.23	0.31	0.37	0.26	0.27	0.2	1				
$k_{\mathrm{11}}$	−0.13	0.35	0.33	0.51	0.31	0.44	0.42	0.43	0.66	0.23	1			
$k_{\mathrm{12}}$	−0.46	0.16	0.11	0.2	0.07	0.08	0.16	0.14	0.16	0.17	0.15	1		
$k_{\mathrm{13}}$	−0.09	0.4	0.41	0.59	0.41	0.7	0.54	0.41	0.55	0.25	0.55	0.16	1	
$k_{\mathrm{14}}$	−0.16	0.38	0.35	0.54	0.27	0.42	0.33	0.44	0.58	0.22	0.66	0.19	0.53	1

According to the correlation coefficient matrix results, all the correlation coefficients were less than or equal to 0.7. Therefore, this dataset has no correlation or collinearity.

##### Statistical description of variables

Table 3 provides a partial description of the statistics for the 14 variables, which facilitates a more intuitive understanding of the data.

**Table 3:** A partial description of statistics for 14 variables.

Variable	K1	K2	K3	K4	K5	K6	K7
Category	0,1	0,1,2	0,1,2	0,1,2,3	0,1,2	0,1,2	0,1,2
Minimum	0	0	0	0	0	0	0
Maximum	1	2	2	3	2	2	2
Mean	0.9743	0.3629	0.3686	1.269	0.3971	0.52	0.7629
Medium	1	0	0	1	0	0	1
Variable	K8	K9	K10	K11	K12	K13	K14
Category	0,1,2	0,1,2	0,1,2	0,1,2	0,1,2	0,1,2	0,1,2
Minimum	0	0	0	0	0	0	0
Maximum	2	2	2	2	2	2	2
Mean	0.95943	1.06	0.1314	0.9657	0.0657	0.9086	0.9286
Medium	0	1	0	1	0	1	1

According to the outcome in Table 3, the median of half of the variables is zero, which means that half of the variables have more than half the values of zero. The rest of the median does not reach 2, indicating that the data matrix is very sparse and corporate carbon information disclosure is fuzzy. Similarly, the mean values of none of the 14 variables’ were higher than half of the range, except for the ninth variable, which barely exceeded 1 (the maximum of the fourth variable was 3; therefore, half of the content should be 1.5). The statistical description of the 14 variables shows that it is necessary to find appropriate methods to study carbon information disclosure and to promote its development.

##### Principal component analysis model

Because the original data did not provide artificial measurement data for carbon information disclosure, we could not make predictions through supervised learning models, such as a linear model, when constructing the formula. Instead, we can only build recipes by using the data themselves through unsupervised learning. It is worth noting that the more pronounced the 14 variables disclosed, the more obvious the carbon information should be disclosed. As the classification variables score each index, the higher the value, the higher the degree of carbon information disclosure. Therefore, the easiest method is to add all the data. However, the contribution of “variables” to carbon information disclosure will differ. Therefore, we need to introduce the concept of variable weight “W.” Principal component analysis (PCA) model describes the weight of each vector (variable) in the matrix through the eigenvalue of the matrix. The more influential the variable, the more significant the proportion of its weight. Therefore, according to the PCA model, we included the weights of 14 indicators in W1–W14. We can then construct a formula for carbon information disclosure:


(3.1) $$Y=\sum_{i=1}^{\mathrm{14}}W_{i}*k_{i}$$

Before calculating the weight k, we need to determine why PCA is appropriate for our research. First, the structure of the dataset satisfies the PCA method. Second, we used KMO and Bartlett’s tests to test whether PCA is good for this dataset. PCA is a good choice if KMO > 0.8, and Bartlett’s test is significant (<0.05). Based on Table 4, we conclude that PCA is a suitable research method.

**Table 4:** KMO and Bartlett’s test.

KMO Test		0.850
Barlett’s test of sphericity	Approx. Chi-square	2078.883
	df	91
	Sig.	0.000

We now need to determine the number of PCs (principal components) we need in our analysis. From Table 5, we should choose a PC whose eigenvalues are greater than 1. Therefore, we should choose the first four PCs and their variance percentages to calculate weight k and the final formulas.

**Table 5:** Variance explained.

Component	Eigenvalues	Variance percentage	Cumulative variance percentage
1	5.563	39.73	39.73
2	1.402	10.02	49.75
3	1.163	8.31	58.06
4	1.069	7.63	65.69
5	0.833	5.95	71.64
6	0.781	5.58	77.22
7	0.632	4.52	81.74
8	0.552	3.94	85.68
9	0.468	3.34	89.02
10	0.411	2.93	91.95
11	0.368	2.63	94.58
12	0.298	2.13	96.71
13	0.278	1.98	98.70
14	0.183	1.30	100.00

Because we have determined that the number of PCs is four, we use the factor loading table or component matrix to calculate the weight k. The outcomes of the factor loading and component matrix in the first four PCs are listed in Table 6.

**Table 6:** Component matrix.

	1	2	3	4
K1	−0.211	0.820	−0.071	−0.080
K2	0.651	0.012	0.020	0.352
K3	0.537	0.012	−0.412	0.304
K4	0.720	−0.009	−0.347	0.295
K5	0.564	0.144	0.480	0.281
K6	0.785	0.211	0.171	0.334
K7	0.653	0.005	0.225	−0.241
K8	0.625	0.068	0.322	−0.482
K9	0.745	0.054	−0.174	−0.328
K10	0.409	0.013	0.530	0.066
K11	0.749	0.010	−0.227	−0.319
K12	0.279	−0.802	0.091	0.021
K13	0.804	0.104	−0.061	0.058
K14	0.727	−0.056	−0.280	−0.248

Then, we divide the values of each PC in Table 6 by the root of the eigenvalues of these PCs and product their ratios by considering only the first four PCs and percentages. Finally, we take the sum of all these new values of PCs to obtain the final values of weight k.

Therefore, we obtain the final formula of carbon information disclosure:


(3.2) $$\begin{array}{c} Y=\sum_{i=1}^{\mathrm{14}}0.0342\times k_{1}+0.2104\times k_{2}+0.1251\times k_{3}\\ +0.1759\times k_{4}+0.2511\times k_{5}+0.2861\times k_{6}\\ +0.1674\times k_{7}+0.1526\times k_{8}+0.1407\times k_{9}\\ +0.1761\times k_{10}+0.1309\times k_{11}-0.0187\times k_{12}\\ +0.2189\times k_{13}+0.1185\times k_{14} \end{array}$$

#### Greenwashing

The other independent variable is greenwashing (GW). The degree of GW has not yet formed a unified measurement standard in academic circles. Referring to the practices of Huang (2020) and Roulet and Touboul (2015), we define GW as selective disclosure and declarative manipulation. The former refers to the selective reporting of environmental issues and the latter refers to the beautification of the corporate image through strategic expression. The final GW degree index was obtained by calculating the geometric mean of the corresponding indices using the two methods. The specific methods are as follows:

Based on Huang et al. (2019), and according to the requirements of relevant laws and regulations, we summarize the issues that enterprises should disclose in social responsibility reports under ideal conditions. We constructed an indicator system of GW degree based on four aspects: governance and structure, process and control, input and output, and compliance, as shown in Table 7. These four aspects contain 20 secondary indicators, which are the content analysis method scores. We manually searched and collected the corresponding content of each secondary indicator in the CSR or sustainable development report. Following this, we (1) determine whether the report discloses the relevant content of indicators and (2) judge whether the disclosure of the enterprise is substantive disclosure or symbolic disclosure. Substantive disclosure is assigned a value of one, symbolic disclosure is assigned a value of zero, and failure to disclose is not recorded. Based on the relevant literature (Clarkson et al., 2008; Walker and Wan, 2012), if enterprises disclose verifiable and inimitable information, such as fact statements, case descriptions, and quantitative descriptions, then their environmental information disclosure is more reliable and belongs to substantive disclosure. By contrast, if the environmental report is mainly a programmatic statement, qualitative disclosure, or a simple copy of the previous year’s statement, which is difficult to verify and easy to imitate, the reliability of the environmental information disclosure is low and belongs to symbolic disclosure.

**Table 7:** Greenwashing measurement index system.

No.	Projects		Symbolic disclosure	Substantive disclosure
1	Governance and institutions	Environmental protection policy and environmental strategy		
2		Objectives and realization of environmental protection		
3		Environmental protection rules, regulations and implementation		
4		Environmental management, organization and operation		
5	Process and control	Environmental certification system and its implementation		
6		Honor and recognition of environmental protection		
7		Investment in environmental protection and comprehensive renovation scheme		
8		Environmental education and training and public welfare activities		
9		Research and development of environmental protection technology and process innovation		
10	Input and output	Energy consumption and reduction measures		
11		Water resources consumption and reduction measures		
12		Greenhouse gas emissions and reduction measures		
13		Emissions and reduction measures		
14		Waste water production and reduction measures		
15		Production and treatment measures of solid waste		
16		Other emission reduction measures, such as greening, noise and logistics		
17	Compliance	Statement of compliance with environmental laws and regulations		
18		Risk assessment brought about by environmental policy		
19		Statement on the impact of industry characteristics on the environment		
20		A statement as to whether a major environmental pollution accident has occurred		

We calculated the total substantive, symbolic, and disclosed information of the sample enterprises to construct selective disclosure indicators (GWS) and expressive manipulation indicators (GWE).

In disclosing the report, the enterprise may have a commitment or performance in some respects, while there is no commitment or performance in others. Therefore, we measure the degree of selective disclosure by comparing the undisclosed items with the total disclosed items. The calculation formula is as follows:


(3.3) Selective disclosure(GWS)=100×(1−Number of disclosed projectsNumber of items tobeannounced)

We use the ratio of symbolic disclosure items to corporate disclosed items to measure the degree of expressive manipulation. The specific calculation formula is as follows:


(3.4) Expressive manipulation(GWE)=100×Number of symbolic disclosuresNumber of disclosed items

According to Huang (2020), GW can be calculated using arithmetic or geometric means. The arithmetic mean is suitable for numerical data. The geometric mean can be applied to quality data. When the total outcome equals the product of all stages (links), the geometric mean can be used to reflect the population’s general level. Since selective disclosure (GWS) is equivalent to quantitative assessment, expressive manipulation (GWE) is equivalent to quality assessment, and the overall situation of enterprise greenwashing is equivalent to the product of quantitative assessment and quality assessment. It is more appropriate to use the geometric mean to theoretically and logically calculate the degree of greenwashing (GW). According to the mean value theorem, the geometric means of several numbers do not exceed the arithmetic mean. When these numbers are equal, the arithmetic mean is equal to the geometric mean: The use of geometric means in this study does not exaggerate the degree of enterprise greenwashing. If significant results can still be observed in this case, the results observed in other calculation methods may be stronger, which helps ensure the robustness of the empirical test results. Therefore, we used the geometric mean to calculate the degree of GW for each enterprise. A large GW value of listed companies represents a higher degree of greenwashing. The formula used is as follows:


(3.5) $$\mathrm{GW}=\sqrt{\mathrm{GWS}\times \mathrm{GWE}}$$

### Control variables

Based on previous studies, we selected control variables, including company size (SIZE), listing age (AGE), debt-paying ability (DEBT), growth ability (GROWTH), operation ability (OPERATION), and industry (IND; Li et al., 2016; Liu et al., 2021). Industry (IND) is a dummy variable. For heavy-pollution enterprises, the value is 1; otherwise, the value is 0. The heavy-pollution industries in this study mainly include coal, mining, textiles, leather, paper, petrochemical, pharmaceutical, chemical, metallurgy, thermal power, and 16 other industries. The definitions of these variables are listed in Table 8.

**Table 8:** Variable definition.

Variables	Symbols	Descriptions
Enterprise value	V	Tobin Q value
Quality of carbon information disclosure	CID	Carbon information disclosure index
Greenwashing	GW	Greenwashing index
Company size	SIZE	Natural logarithm of total assets at the end of the period
Listing age	AGE	Number of years an enterprise has been listed
Debt paying ability	DEBT	Current ratio
Development ability	GROWTH	Sustainable growth rate
Operation ability	OPERATION	Total asset turnover
Industry	IND	Heavy pollution enterprises are recorded as 1, while other enterprises are recorded as 0
Year	YEAR	Year virtual variable

### Model design

According to the above correlation analysis, the following three models were designed to test H1, H2, and H3, and the following three models were designed, respectively:


$$\begin{array}{c} V=\beta_{0}+\beta_{1}CID+\beta_{2}SIZE+\beta_{3}AGE+\beta_{4}DEBT+\beta_{5}GROWTH\\ +\beta_{6}OPERATION+\beta_{7}IND+\beta_{8}YEAR+\varepsilon \end{array}$$

                   Model (1)


$$\begin{array}{c} V=\beta_{0}+\beta_{1}CID+\beta_{2}GW+\beta_{3}CID\times GW+\beta_{4}SIZE+\beta_{5}AGE\\ +\beta_{6}DEBT+\beta_{7}GROWTH+\beta_{8}OPERATION+\beta_{9}IND\\ +\beta_{10}YEAR+\varepsilon \end{array}$$

                   Model (2)


$$\begin{array}{c} V_{it+2}=\beta_{0}+\beta_{1}CID_{it}+\beta_{2}GW_{it}+\beta_{3}CID_{it}\times GW_{it}\\ +\beta_{4}SIZE_{it}+\beta_{5}AGE_{it}+\beta_{6}DEBT_{it}+\beta_{7}GROWTH_{it}\\ +\beta_{8}OPERATION_{it}+\beta_{9}IND_{it}+\beta_{10}YEAR_{t}+\varepsilon_{it} \end{array}$$

                   Model (3)

Model (1) was used to test H1 and the direct impact of carbon information disclosure quality on enterprise value. Model (2) was used to test H2, namely, the moderating effect of greenwashing behavior on carbon disclosure on enterprise value in the short run. Model (3) was used to test the moderating effect of greenwashing behavior on carbon disclosure on enterprise value in the long run (H3). This study verified H2 and H3 by introducing the cross-multiplication term of CID × GW. We also centralize the sub-variables used by the cross-product term before applying Models (2) and (3) to avoid multicollinearity caused by the cross-product term affecting the accuracy of the statistical test results and then multiply them to obtain the cross-product term, followed by regression.

## Results

### Descriptive statistics

Table 9 presents descriptive statistics for each variable. The mean enterprise value (V) for the 350 samples was 1.690, and the standard deviation was 1.401. Among the sample enterprises, the carbon information disclosure of the quality evaluation index (CID) was 4.216 at the highest and 0.0342 at the lowest, while the mean value was 1.452. This indicates that the carbon information disclosure level of the sample companies is generally low, and the quality difference in carbon information disclosure is significant. The greenwashing (GW) degree was 97.47, 0, 45.68, and 26.50, indicating that the difference in the degree of greenwashing of the sample companies is substantial and the fluctuation is strong. The difference in the top and minimum values in listing age (AGE) was 26, indicating a specific difference between the sample corporate listing years. The minimum value of debt-paying ability (DEBT) was 0.175, the maximum value was 10.57, and the standard deviation was 1.143.

**Table 9:** Descriptive statistics.

Variables	N	Mean	SD	Minimum	Maximum
V	350	1.690	1.401	0.698	16.423
CID	350	1.452	1.112	0.0342	4.216
GW	350	45.68	26.50	0	97.47
SIZE	350	24.82	1.484	21.64	27.90
AGE	350	16.99	5.221	1	27
DEBT	350	1.577	1.143	0.175	10.57
GROWTH	350	0.0841	0.0701	−0.181	0.763
OPERATION	350	0.695	0.449	0.0890	3.096
IND	350	0.243	0.429	0	1

The minimum value of growth ability (GROWTH) was 0.181, the maximum value was 0.763, and the standard deviation was 0.0701. The gap in the debt-paying ability of the sample companies is noticeable, but the opening of growth ability is small. Some enterprises have strong solvency and developmental abilities. The average value of industry (IND) was 0.243, indicating many non-heavy polluting companies in the sample companies. Overall, most data were relatively stable.

### Correlation analysis

Table 10 shows the Pearson correlations for all the dependent, independent, and control variables. The table shows that CID is negatively correlated with V, which is insignificant and different from the expected result. The relationship between the two needs to be further tested using regression analysis. As expected, there is a positive correlation between greenwashing (GW) and enterprise value, and this is particularly significant. Company size (SIZE) is negatively correlated with enterprise value. This indicates that enterprises with more total assets in the sample companies have a smaller relative value. Debt-paying and growth abilities are significantly and positively correlated with enterprise value. This indicates that the better the solvency of the enterprise, the higher the sustainable development capacity, and the higher the value of the enterprise. In addition, there was a strong correlation between several control variables, and no correlation coefficient exceeded 0.6. Table 11 shows that the variance inflation factor (VIF) value is less than 2 and the 1/VIF value is more significant than 0.6, indicating no multicollinearity problem.

**Table 10:** Correlation analysis.

Variable	V	CID	GW	SIZE	AGE	DEBT	GROWTH	OPERATION	IND
V	1.0000								
CID	−0.0650	1.0000							
GW	0.091*	−0.517***	1.0000						
SIZE	−0.465***	0.324***	−0.109**	1.0000					
AGE	−0.0160	0.0420	0.0300	−0.229***	1.0000				
DEBT	0.435***	−0.199***	0.0860	−0.458***	0.0530	1.0000			
GROWTH	0.261***	−0.0720	0.0540	−0.127**	0.0020	0.284***	1.0000		
OPERATION	0.0150	0.0310	0.0770	−0.127**	0.0290	0.0330	0.100*	1.0000	
IND	0.261***	0.177***	−0.0850	−0.164***	0.193***	0.211***	0.0810	−0.0310	1.0000

*** denotes significance at the 1% level, ** denotes significance at the 5% level, and * denotes significance at the 10% level.

**Table 11:** Variance inflation factor test for variables.

Variable	VIF	1/VIF
CID	1.24	0.806230
SIZE	1.51	0.660300
AGE	1.20	0.834137
DEBT	1.41	0.711037
GROWTH	1.11	0.900680
OPERATION	1.04	0.961556
IND	1.17	0.857184
Mean VIF	1.40	

### Regression results

The regression results are presented in Table 12. The first column shows the test of a model (1). The main regression results show that CID is positively correlated with an enterprise value at a 5% significance level. This indicates that the higher the CID level, the higher the enterprise value. Carbon information disclosure improves enterprise value. This result confirms H1 and is consistent with the results of previous studies. It is good for carbon disclosure investors to consciously and actively take responsibility for a good corporate image, increase enterprise reputation, and thus bring about enterprise capital premiums. This will attract more investment and carbon disclosure to alleviate enterprises’ and investors’ information asymmetry. It will improve investors’ recognition and trust in the enterprise, stimulate stock purchase intention, and further promote the value creation of enterprises. In addition, SIZE is significantly negatively correlated with enterprise value. The greater the total assets of a company, the lower its relative value. The coefficients of DEBT and GROWTH are significantly positive. This indicates that the higher the enterprise’s debt-paying and sustainable development abilities, the higher the enterprise value.

**Table 12:** Multiple regression results.

Variables	(1)	(2)	(3)
	V	V	V_t + 2_
CID	0.105**	0.255***	0.178
	(2.34)	(3.36)	(1.40)
GW		0.010***	0.008
		(3.14)	(1.44)
CID×GW		0.005**	0.005
		(2.18)	(1.09)
SIZE	−0.383***	−0.402***	−0.388***
	(−9.26)	(−8.02)	(−4.93)
AGE	−0.048***	−0.051***	−0.054***
	(−4.24)	(−4.07)	(−2.66)
DEBT	0.250**	0.237***	0.218**
	(2.51)	(3.78)	(2.39)
GROWTH	3.216***	3.047***	2.922**
	(3.19)	(3.37)	(2.22)
OPERATION	−0.148**	−0.215	−0.228
	(−2.14)	(−1.56)	(−1.10)
IND	0.512***	0.524***	0.556**
	(3.06)	(3.46)	(2.32)
YEAR	controlled		
Constant	11.039***	11.899***	11.424***
	(9.83)	(8.64)	(5.32)
Observations	350	350	210
R-squared	0.363	0.382	0.291

T-values are in parentheses. *** denotes significance at the 1% level, ** denotes significance at the 5% level.

Column 2 of Table 12 presents the results for H2. In the test of Column 2, the coefficient of CID × GW between the quality of carbon disclosure and greenwashing is 0.005, which is significantly positive at the 5% level, indicating a positive regulatory effect in the short run. Hence H2 is verified. Compared with the study by Li et al. (2017a, 2017b), we supplement the role of GW in the relationship between CID and enterprise value. In the short run, greenwashing might not be detected and enterprise value might be improved because of “green manipulation.” In particular, for impression management in the voluntary disclosure of environmental information of a floating green enterprise, carbon disclosure responsibility fulfillment will be more active, carbon performance will be better, and transparent information can attract more investors, further enhancing the positive effect of carbon disclosure on enterprise value. In the short run, GW plays a positive moderating role in the relationship between carbon information disclosure and enterprise value. The coefficients of the other variables are consistent with the main regression.

Column 3 of Table 12 shows the test for H3. The coefficient of CID × GW is 0.005, which is not significant, indicating that greenwashing has no significant positive regulatory effect on the relationship between CID and V after 2 years. The positive effect of greenwashing fades in the long run. Consumers may perceive enterprises’ pseudo-social responsibility behavior in the long run, and brand loyalty and corporate reputation may be adversely affected. This damages consumers’ confidence affects investor sentiment, and causes the promotion effect of carbon information disclosure on enterprise value to fade. The results of the other control variables are consistent with the previous results.

### Endogeneity tests

High-quality carbon information disclosure will improve corporate value and vice versa; high corporate value may also have an impact on the quality of carbon information disclosure. To avoid estimation bias caused by bidirectional causality, instrumental variables are constructed, and two-stage regression estimation (2SLS) is used to control the endogeneity problem. Referring to Larcker and Rusticus (2010) and Yan et al. (2020), the instrumental variable is represented by the average carbon information disclosure quality (IVCID) of the province where the sample is located, which is related to CID but is less likely to directly affect the value of a single enterprise. In the first regression stage, control variables and tools (IVCID) were taken as independent variables, and carbon disclosure quality (CID) was taken as the dependent variable. The fitting value or predictive variable (PCID) was then obtained. In the second-stage regression, predictive variables (PCID) and control variables were used as independent variables to conduct a regression of enterprise value (V). Shea’s partial *R*^2^ value was used to test the strength of the tool variable. The results show that Shea’s partial *R*^2^ is 0.1896, the *F*-statistic is 102.67, and the *p*-value is 0.0000. Therefore, the selected tool variable was a strong tool variable. Table 13 shows that the regression coefficient of the enterprise value (V) and the prediction variable (PCID) is 0.193, which is significant at the 5% level. From this, we can see that the two variables have a significant positive correlation, which is consistent with the above results.

**Table 13:** Endogeneity tests.

Variables	First stage	Second stage
	CID	V
IVCID	0.876^***^ (8.89)	
PCID		0.193^**^ (2.27)
SIZE	0.154^***^ (3.84)	−0.405^***^ (−9.23)
AGE	−0.0103 (−0.99)	−0.0490^***^ (−4.37)
DEBT	−0.0574 (−1.13)	0.258^***^ (2.61)
GROWTH	−1.001 (−1.36)	3.247^***^ (3.28)
OPERATION	0.230^**^ (2.08)	−0.168^**^ (−2.38)
IND	0.585^***^ (4.91)	0.456^***^ (2.66)
YEAR	controlled	
Constant	−3.615^***^ (−3.37)	11.50^***^ (9.98)
Observations	350	350
*R*-squared	0.347	0.359

*** denotes significance at the 1% level, ** denotes significance at the 5% level.

### Robustness tests

We conducted a number of analyses to ascertain the robustness of the results. First, considering the possible heteroscedasticity of the samples, this study adopts the weighted least squares method to perform regression on the basis of the previous regression, as shown in Column 1 of Table 14. The regression results verify Hypothesis 1. Second, we use another Tobin’s Q formula [market value A / (total assets − net intangible assets − net goodwill)] to replace the previous formula [market value B / (total assets − net intangible assets − net goodwill); Yan et al., 2020]. Then, we obtain another Tobin’s *Q* value (VB) and carry out the regression test again to avoid the instability of empirical results due to the different calculation methods of market value. As shown in Columns 2, 3, and 4 of Table 14, the research results are consistent with the previous results. Third, following Tian et al. (2021), to further test the robustness of the results, we randomly selected 70% of the total sample and re-estimated the main regression. As shown in columns 5, 6, and 7 of Table 14, the results are consistent with the baseline results. Therefore, our baseline findings were robust and reliable.

**Table 14:** Robustness tests.

Variables	(1)	(2)	(3)	(4)	(5)	(6)	(7)
	V	VB	VB	VB_t + 2_	V	V	V_t + 2_
CID	0.116*** (4.12)	0.091** (2.11)	0.236*** (3.18)	0.155 (1.24)	0.143** (1.99)	0.395*** (3.50)	0.220 (1.09)
GW			0.009*** (3.10)	0.008 (1.45)		0.012*** (2.97)	0.007 (0.92)
CID×GW			0.005** (2.29)	0.004 (1.05)		0.009*** (2.75)	0.006 (0.90)
SIZE	−0.274*** (−11.35)	−0.343*** (−8.51)	−0.361*** (−7.36)	−0.368*** (−4.74)	−0.486*** (−7.78)	−0.507*** (−7.79)	−0.519*** (−5.04)
AGE	−0.024*** (−4.08)	−0.043*** (−4.19)	−0.046*** (−3.71)	−0.058*** (−2.88)	−0.088*** (−4.89)	−0.089*** (−4.78)	−0.102*** (−3.42)
DEBT	0.084* (1.71)	0.280*** (2.83)	0.267*** (4.36)	0.245*** (2.73)	0.420*** (2.78)	0.399*** (4.81)	0.365*** (2.99)
GROWTH	2.299*** (5.14)	3.042*** (3.24)	2.872*** (3.25)	2.303* (1.78)	2.320** (2.37)	2.287** (2.05)	2.564 (1.55)
OPERATION	−0.093** (−2.08)	−0.174** (−2.55)	−0.238* (−1.76)	−0.226 (−1.10)	−0.449*** (−3.33)	−0.443** (−2.15)	−0.544* (−1.66)
IND	0.128 (1.53)	0.510*** (3.15)	0.524*** (3.54)	0.557** (2.36)	0.668*** (3.81)	0.585*** (2.98)	0.721** (2.33)
YEAR	controlled						
Constant	8.284*** (12.30)	9.869*** (9.14)	10.690*** (7.94)	10.932*** (5.17)	13.968*** (8.53)	14.947*** (8.35)	15.236*** (5.41)
Observations	350	350	350	210	245	245	147
*R*-squared	0.368	0.359	0.378	0.290	0.421	0.447	0.369

*** denotes significance at the 1% level, ** denotes significance at the 5% level, and * denotes significance at the 10% level.

### Further analysis

#### Industry heterogeneity test

Heavy-pollution enterprises are resource-intensive with long production processes that require a large amount of energy and resources and discharge more pollution. Therefore, heavy-pollution enterprises should focus on a “double carbon” target. In this study, sample enterprises were divided into heavy-pollution enterprises and non-heavy-pollution enterprises, and regression was conducted to investigate the influence of industry heterogeneity on the relationship between CID and enterprise value. Table 15 presents the results. Columns 1 and 2 show the influence of CID on heavy-pollution enterprises and non-heavy-pollution enterprises on enterprise value. The results show that the coefficient of CID is 0.033 for heavy-pollution enterprises, which is not significant. The CID coefficient is 0.111 for non-heavy-pollution enterprises, positively correlating with an enterprise value at the 1% level. The positive impact of the CID level on the enterprise value of non-heavy-pollution enterprises is more obvious than that of heavy-pollution enterprises. This is the same as Yan and Chen’s (2017) conclusion. Listed companies consider costs and benefits when managing carbon emissions. Heavy-pollution enterprises need to consume a large amount of energy in production, and they also need to confirm future environmental liabilities. Additionally, non-heavy-pollution enterprises face relatively little environmental pressure during normal production activities. As a result, they are more sensitive to the government’s ecological regulation policies and pay more attention to CID than heavy-pollution enterprises. This results in a more significant improvement in the value. The regression results for the other control variables are similar to those of the full-sample regression results.

**Table 15:** Further analysis.

Variables	Industry heterogeneity test		Legal environment heterogeneity test	
	Heavy polluting enterprise	Non-heavy polluting enterprise	Good legal environment	Poor legal environment
	V	V	V	V
CID	0.033 (0.13)	0.111*** (3.49)	0.137** (2.05)	0.104 (1.66)
SIZE	−0.577** (−2.28)	−0.362*** (−8.63)	−0.429*** (−5.46)	−0.385*** (−7.96)
AGE	−0.184 (−1.61)	−0.036*** (−4.22)	−0.044*** (−2.61)	−0.062*** (−3.63)
DEBT	0.362*** (2.69)	−0.078 (−1.17)	0.302** (2.09)	0.176 (1.62)
GROWTH	2.635 (1.44)	2.519*** (3.99)	2.955*** (2.77)	2.913* (1.91)
OPERATION	−0.580*** (−2.75)	−0.028 (−0.39)	−0.311** (−2.22)	0.044 (0.50)
IND			0.511** (2.44)	0.415* (1.68)
YEAR	controlled			
Constant	18.498** (2.50)	10.841*** (8.92)	12.136*** (5.71)	11.309*** (8.99)
Observations	85	265	220	130
*R*-squared	0.426	0.332	0.366	0.434

*** denotes significance at the 1% level, ** denotes significance at the 5% level, and * denotes significance at the 10% level.

#### Legal environment heterogeneity test

In China’s economic environment, in addition to internal enterprise factors, the institutional environment also plays a profound role in influencing enterprises’ behavior (Williamson, 2000). Wang et al. (2018) compiled the NERI Index of Marketization of China’s Provinces’ 2018 Report to measure the degree of marketization. The sub-indexes of the Chinese provinces included in this report can be used as substitute variables to study institutional differences among provinces in the Chinese market (Gupta et al., 2022). According to Liu and Zhang (2017); Xie (2017), and Gupta et al. (2022), the legal system environment index in the NERI index was selected to conduct heterogeneity research. The higher the index, the better the legal environment of the region. According to the index, enterprises in regions larger than the mean are divided into the good legal environment group, and those in regions smaller than the mean are divided into the poor legal environment group. The regression results are presented in Table 15 and Columns 3 and 4. It can be seen that the direct impact of CID on the enterprise value is more significant in the good legal environment group than in the poor legal environment group. In other words, CID plays a more significant role in enhancing enterprise value in a good legal environment. The legal environment in which an enterprise is located may affect the profit and loss of some specific behaviors of the enterprise, thus affecting the motivation and decision preferences of the enterprise. Companies in a better legal environment may have stronger incentives to disclose higher-quality carbon information. Moreover, a sound legal regulatory environment can improve enterprise performance and effectively promote economic growth (La Porta et al., 2000). Therefore, in a good legal environment, enterprises can actively disclose carbon information and increase their ability to create value.

## Conclusion, revelations, and limitations

### Conclusion

This study explores the impact of carbon disclosure on enterprise value and the moderating effect of greenwashing behavior on these two factors. Based on the data of the constituents of the Social Responsibility Index of the China Shanghai Stock Exchange from 2016 to 2020, the innovative CID index measurement formula was obtained through the data analysis of the specific characteristics and factors of the carbon information in the corporate social responsibility report or sustainable development report and PCA. The following conclusions were drawn from the regression analysis of carbon information disclosure, greenwashing behavior, and enterprise value. (1) A good CID can improve enterprise value. (2) GW can promote the improvement of corporate value through CID to a certain extent in the short run. In the long run, this promotion effect fades. (3) The positive impact of the CID level on the enterprise value of non-heavy polluters is more obvious than that of heavy polluters. (4) The positive impact of the CID level on enterprise value is more obvious for enterprises in a good legal environment than for enterprises in a poor legal environment.

### Revelations

This study has several implications for managers and policymakers. First, China has not yet issued relevant laws and regulations on how and which carbon information the enterprises should disclose. Hence, enterprises’ carbon information is not standardized, scattered, and even produces greenwashing behavior. Information users must spend considerable energy, time, and cost to extract effective carbon information. Greenwashing can bring some benefits to enterprises in the short term. However, in the long term, greenwashing does not add value to enterprises. Greenwashing is a type of information whitewashing with an unacceptable negative activity. Greenwashing seriously damages the interests of investors, and once exposed, has serious adverse effects on enterprises. The government should therefore establish relevant standards for mandatory CID based on China’s reality, and regulate enterprises’ information disclosure behaviors. In addition, relevant departments should strengthen the supervision of enterprises across different industries and improve the quality of the legal environment in various regions. They should improve the effectiveness of the capital market and narrow the value gap between CID and enterprise value across different industries and legal environments.

The enterprise should establish a special carbon reduction risk monitoring mechanism and set up low-carbon management to strengthen the management of low carbon. This can effectively improve the production efficiency and operations of enterprises; reduce costs; set up a good corporate image and corporate reputation; strengthen investors’, creditors’, and consumers’ trust; and boost the sustainable development of the enterprise. Management psychology believes that changing unreasonable and incorrect behaviors can transform negative factors into positive ones. Therefore, enterprises should also strengthen the supervision of CID to prevent management from disclosing false carbon information for their selfish interests that result in the greenwashing behavior of enterprises. Enterprise managers need to correctly understand the negative effects of greenwashing behavior and the significance of CID in the long-term development of enterprises. The disclosure of environmental information should be timely, accurate, and comprehensive.

### Limitations

There are some limitations to this study. When constructing the CID formula in this study, the choice of variables was subjective, and there were more potential variables affecting CID. There are also interactions between the variables, which warrant further study. Additionally, our sample is limited to Social Responsibility Index on the Shanghai Stock Exchange. Future research can be based on further perfecting the carbon disclosure formula, selecting a more extensive social responsibility report or sustainable development report, and expanding the research scope, not restricting it to a particular area, region, or country. Additionally, as relevant government departments pay more attention to GW, more methods can be selected to conduct in-depth research on the influence of policy regulations on GW and the adverse consequences of GW in enterprises in the future.

## Data availability statement

The original contributions presented in the study are included in the article/Supplementary material, further inquiries can be directed to the corresponding author.

## Author contributions

QC conceptualized and designed the empirical research and wrote this manuscript. YZ designed the empirical research, collected the data, and wrote this manuscript. HD designed the empirical research and performed the data analysis. MR and WZ performed the data analysis and revised the manuscript. All authors contributed to the article and approved the submitted version.

## Funding

The work described in this manuscript was supported by the Sichuan Soft Science Research Project (2019JDR0345) and the Social Science Planning Project of Sichuan Province (SC19TJ026).

## Conflict of interest

The authors declare that the research was conducted in the absence of any commercial or financial relationships that could be construed as a potential conflict of interest.

## Publisher’s note

All claims expressed in this article are solely those of the authors and do not necessarily represent those of their affiliated organizations, or those of the publisher, the editors and the reviewers. Any product that may be evaluated in this article, or claim that may be made by its manufacturer, is not guaranteed or endorsed by the publisher.
